# Some Contributions to the Stastistics of the Operation of Nephrotomy, So-called, with a Few Observations Thereon

**Published:** 1873-02

**Authors:** Thos. H. Kearney

**Affiliations:** Professor of the Principles of Surgery and Surgical Pathology in the Miami Medical College


*Some Contributions to the Stastistics of the Operation of Nephrotomy, so-called, with a few Observations thereon.* By Thos. H. Kearney, M.D., Professor of the Principles of Surgery and Surgical Pathology in the Miami Medical College.

There are certain diseases and operations, which, in consequence of their infrequency, appear to fade out of the professional memory, at times. Such is the operation of nephrotomy, which, though advocated in the Hippocratic writings, and commented on by many of the ancient writers subsequent to the time of Hippocrates, is at the present day hardly mentioned by surgical authors. Mr. Erichsen (in the 5th edition of his work), Sir Wm. Fergusson, Professor Hamilton, and Mr. Gant, do not mention it at all. Dr. Ashurst is very brief on the subject, and does not give any statistical information beyond a bare reference to the case of Marchetti. Professor Gross, in the edition of his work just issued, devotes eleven lines to the subject, and cites three cases of the operation, so-called: that of Marchetti, and those of Gunn, of Chicago, and Durham, of Guy's Hospital. These last two, it will be seen by reference to the published accounts in the journals, were merely exploratory operations, by which the kidney was exposed, so as to admit of being examined for calculus, according to the suggestion of Mr. Thos. Smith. Professor Gross might have correctly mentioned the case of Mr. Bryant, of Guy's, in which the kidney was incised, but without the discovery of the suspected stone. That complete nephrotomy was performed in this case was proved at the *post-mortem* examination of the organ.

During a recent discussion in the Academy of Medicine, the question of the infrequency of such operations as the one then reported, came up; and, in consequence, the writer was induced to make some researches on the subject. The following extracts are given *verbatim*, or as nearly in literal accordance with the original as possible.

Chelius uses the following language: "The removal of the stone by *cutting* (nephrotomia) can only be undertaken, when an œdematous, or fluctuating swelling, or a fistula, has formed in the loins. Having opened the abscess, its bottom must be examined with the finger or the probe, and if a stone be met with, it must be removed, after enlarging the wound, if it be too confined.—*Syst. Surgery*, Am. ed., vol. 3, p. 268.

After describing pyelitis from calculus and other causes, Sir Henry Thompson adds: "*Abscesses* may be formed under these circumstances; also as the sequel of nephritis; they should not be opened until the tumor points and the diagnosis is perfectly clear. Often they are perinephritic, although originated by disease within the kidney itself; occasionally a calculus may be removed through the opening made."—*Holmes' Syst. Surgery*, vol. 4, p. 333, 1st ed.

Velpeau, after expressing his incredulity in regard to the operation of true nephrotomy having ever been attempted, goes on to say: "The operation cannot, in reality, be proposed, except in a small number of cases, as in those in which the flank, which has become the seat of an evident fluctuation, after the existence of various signs of calculous affections in the kidney, would enable us to reach the morbid collection with facility and certainty," etc., etc. "In such cases the operation is so simple, and is reduced to so small a matter, and has moreover, to be modified by so many controlling circumstances, that it would be useless to describe it in detail."—*Mott's Velpeau*, Blackman's ed., vol. 3, p. 692.

After making some admirable remarks in reference to the ambiguity of symptoms, and the difficulties and dangers of the operation, Mr. Benjamin Bell condemns the performance of true nephrotomy; but adds: "When, indeed, the inflammation induced by a stone in the kidney terminates in an abscess, and when the matter thus collected forms a tumor, in which a fluctuation is distinguished, little or no danger can ensue from laying it open: and in such an event, the stone that produced the tumor will either be discharged along with the matter, or it may, if it can be laid hold of, be afterward taken out with safety."—*Syst. Surg.*, vol. 6, p. 216, 7th ed.

"The incision, in parallelism with the sacro-lumbalis muscle, must be free, and sufficiently deep to enable the surgeon to explore with his finger the extent of the cavity, and seek for gravel, or any large concretion."—*Cyclop. Pract. Surg.*, art. Nephrotomy.

Samuel Cooper, in his dictionary of Practical Surgery, uses these words, under the head of Nephrotomy: "When a stone, from its size, cannot pass from the kidney, and excites inflammation and suppuration, no doubt the surgeon may make an incision into the tumor, and extract the calculus. In this sense, nephrotomy is certainly a practicable operation. Warner contends that it can only be practiced in such circumstances, notwithstanding whatever may have been said by Marchetti, or others, upon the subject. In such a case, the operation would not be attended with any greater difficulty than the opening of an abscess in any other part of the body."

This last sentence is almost a verbatim quotation from Joseph Warner's Cases in Surgery, p. 241.

It is evident from the foregoing extracts, that the authors quoted do not regard such an operation as the opening of a lumbar abscess, in connection with a disorganized kidney, and the extraction of a renal calculus, an operation of any magnitude; and it may be inferred from the language used, that such cases are not exceedingly rare. For none of them comment on their infrequency, but speak of the removal of a calculus under such circumstances as a matter of course. Yet, an examination of the literature of the subject, as far as rather limited opportunities would permit, affords only the following cases—sufficient, however, to dispose of the claim made for such a case recently, that it was the second of its kind reported!

Cases.—A case is referred to by Heister, though without the particulars. After arguing in favor of the practicability and propriety of true nephrotomy in certain cases, he proceeds in the following quaint language:

"But nothing can be more reasonable than to perform Nephrotomy, when we are directed to it by Nature pointing out the place, by a Tumour and Abscess formed in the loins, from a Calculus in the Pelvis or Kidney. In such a Case, we are also supported by the Advice and Authority of Schenckius, Wedelius, and Meekren; together with Lavaterus, formerly an eminent Physician and Surgeon of Helvetia, with whom I amicably cohabited for some time, in the Year 1710, he then practicing Surgery at London with great Applause. He at that time told me that he had not only performed this Operation with Success in the above-mentioned Case, but had also publickly declared (in the last Page but one of a Treatise published in the Year 1708, at Utrecht on the Rhine, *de Atriteis et Hyposspadiceis*), 'I perform the Operation of Nephrotomy, on either of the Kidneys, when Nature directs to that Practice by forming an Abscess.'"—*A General System of Surgery*, by Lawrence Heister, vol. 2, p. 163.

"Gaspard Bauhin operated on a girl, born of parents of calculous diathesis, who was attacked with a tumor in the lumbar region, following a total suppression of urine. A surgeon applied poultices to this tumor, during two months, in the hopes that it would suppurate, but without any success. At last he distinguished a very hard point in the tumor, into which he made an incision through which he extracted two calculi. This operation was followed by all possible success."—*Rayer*, *Maladies des Reins*, vol. 3, p. 59.

"Rousset also reports that a man who had suffered for a long time with nephritic pains, accompanied by vomiting, presented afterwards a considerable tumor between the groin and iliac bone. A very experienced surgeon made an opening into the tumor and evacuated a quantity of urine mixed with pus, and a calculus of the size of a bean.—*Ibid.*, vol. 3, p. 222.

"Aymar, a surgeon of Grenoble, communicated to Riviere the case of a man who had a tumor in the lumbar region, from which he removed calculi as large as almonds, and later one the size of a bean. During ten years, consecutively, there ran out of this fistula a serous fluid, which would soak the linens applied to it, so that it seemed as if they had been dipped in water. The fistula closed from time to time, only to open again spontaneously after the lapse of some months."—*Ibid.*, vol. 3, p. 325.

"Roonhuysen extracted, by opening an abscess in the right kidney, a tolerably large stone, of which he gives a drawing in his work. He conducted the treatment of the wound, according to the rules of the art, to a perfect cure, so that the patient lived in perfect health for two years after. At the end of this time, a new inflammation occurred on the same side of the loins. The surgeon, not doubting but that there was still some foreign body there, opened the cicatrix and removed a second stone, smaller than the first. The wound closed, and the patient since has always enjoyed good health."—*Ibid.*, vol. 3, p. 226.

"Ledran reports a case of abscess in the loins, after opening which, he extracted a calculus as large as a pea."—*Ibid.*, vol. 3, p. 233.

"Collot saw Cresse incise a lumbar abscess, from which he removed a stone."—*Ibid.*, vol. 3, p. 225.

"Mr. Gregory Smith has seen three cases of abscess in the kidney, resulting from the presence of calculi in the organ. One of these cases was that of a patient who was admitted some years since, in a state of hectic, into St. George's Hospital. There was a large abscess pointing, and apparently about to burst in the lumbar region. Sir B. Brodie laid the abscess freely open, and on introducing his finger into the cavity, detected several loose bodies at the bottom of it; these, being removed by means of a small forceps, proved to be three calculi of the size of nutmegs."—*London Lancet*, 1839 and 1840, vol. 2, p. 449.

T. Spencer Wells makes the statement: "I have twice opened perirenal abscesses in the loin, and in one case removed a small renal calculus through the opening."—*Dub. Quarterly Jour.*, Feb. 1867.

Nelaton, in the fifth volume of his *Elemens de Pathologie Chirurgicale*, records a case of the removal of a large renal calculus from an abscess, the opening of which was accomplished partly by incision, and partly by caustic.

Thus, we find eleven cases recorded, in which abscesses communicating with disorganized kidneys, and containing calculi, have been opened and the calculi immediately extracted. Others have been reported, where the abscesses have been incised and the calculi removed, after varying intervals; but are omitted here, as not being cases in point. Such are the cases of Lafitte and Pouteau.

Another point raised in the recent discussion in the Academy, was as to the proper application of the term nephrotomy—the compiler of this paper taking the ground, that, although surgical writers generally sanction the use of the word in its widest sense, yet, as precision of meaning is so important in the statement of scientific facts, the word nephrotomy ought to be used only in its literal sense. As now used, the word is absolutely worthless for conveying any definite meaning; for under it we find indexed throughout surgical literature such cases as the opening of perirenal abscesses, the dilatation or enlargement of renal fistulæ, the extraction of calculi from abscesses, as in the cases quoted, and the total extirpation of the organ, as well as incisions into the true substance of the gland—the only cases to which the word ought to be applied. Perhaps the only value the word now possesses is in the convenience it affords for foisting on the profession an insignificant operation, under the dignified and high-sounding name of nephrotomy!

The writer is happy in being able to quote so high an authority as Rayer in support of the opinion he advanced on this point. The following extracts from that author's work show very satisfactorily his disapproval of the loose and vague use of the word, which includes, under nephrotomy, operations varying so widely in character.

"Lafitte and Pouteau have practiced with success in similar conditions (that is, when an abscess exists which has had its origin in the kidneys), the opening of such abscesses. Not only have they been so fortunate as to effect a sensible improvement in the health of the patients after the evacuation of the pus, but also to extract the calculi which had been the cause of the inflammation. This operation has been followed by a complete cure. Nevertheless, I must remark that they have erroneously given the name of nephrotomy to the simple opening of an extra-renal abscess, the result of calculus pyelitis."—*Rayer*, vol. 3, p. 52.

This sentence occurs introducing the account of the operation of Hippeau:

"Since then" (that is, the time when Hevin wrote upon nephrotomy, in the *Memoires de l'Academie Royale de Chirurgie*), "there have been several times cited, as examples of nephrotomy, cases of simple incision of abscesses of the loins proceeding from the kidneys. Such is the following reported by Hippeau, etc."—*Ibid.*, p. 236.

"Several observations of lumbar abscesses proceeding from the kidneys, opened either with a cutting instrument or caustic, and whence renal calculi have been removed, have been also reported by Lafitte, and quoted erroneously as examples of nephrotomy." —*Ibid.*, p. 235.

After stating the opinions of various of the older surgeons in regard to the advisability of the operation of nephrotomy, Rayer continues thus: "I take this occasion to remark, that although the question of *nephrotomy* had been disputed, there was almost a perfect unanimity among surgeons and physicians of the highest authority, recommending the opening of lumbar abscesses proceeding from the kidneys, in order to give issue to pus and sometimes even to calculi."—*Ibid.*, p. 222.

The foregoing quotations and remarks represent, substantially, the writer's comments and criticisms on the case reported in the Academy on the 26th October; and are directed solely to the statements then given of the operation and the amplification of those statements called out by the remarks then made. It is not intended to follow that case through its subsequent variations of history.

*My remarks were intended to apply to a case of lumbar abscess, resulting from chronic suppuration and degeneration of the kidney, caused by the presence of a calculus; and such a case as the original report made it appear to be.—Lancet and Observer.*

